# Capillaroscopic findings in children and adolescents with raynaud’s phenomenon: results from study in 92 patients

**DOI:** 10.1186/1546-0096-12-S1-P283

**Published:** 2014-09-17

**Authors:** Berta Lopez Montesinos, Maria Isabel Gonzalez Fernandez, Laura Fernandez Silveira, Inmaculada Calvo Penades

**Affiliations:** 1 Pediatric Rheumatology Unit, Hospital La Fe, Valencia, Spain

## Introduction

Raynaud’s phenomenon (RP) appears to be underestimated in the pediatric population and its prevalence is unknown. However, RP is the earliest and the most common clinical manifestation of diffuse connective tissue diseases.

Nailfold capillaroscopy is an easily performed, non-traumatic and low cost technique, with a confirmed role in discrimination between primary and secondary RP, playing an important role in the assessment of autoimmune rheumatic diseases.

## Objectives

Identify all patients registered as RP in a Pediatric Rheumatology Unit. Describe the demographic and clinical features of these patients. Assess nailfold capillaroscopy in these children and adolescents with RP and the relation of the clinical features to the capillaroscopic pattern.

## Methods

Medical records (2003-2013) from patients with RP followed in our Pediatric Rheumatology Unit were reviewed for demographic data, familial history, trigger factors, Raynaud pattern, clinical manifestations, associated conditions and auto-antibodies positivity. Capillaroscopic patterns were defined as normal (NP), nonspecific pathological (NPP) and specific pathological (SPP). Capillaroscopy was performed, distinguishing primary RP (PRP), secondary RP (SRP) and undifferentiated RP (URP). Associated autoimmune rheumatic diseases: systemic sclerosis (SSc), juvenile dermatomyositis (JDM), systemic lupus erythematosus (SLE), mixed connective tissue disease (MCTD).

## Results

101 patients with RP, F:M 84:16. Age at onset 10,42 +/- 3,70 years old. RP classification: 28% PRP, 35% SRP, 37% URP. Familial history: 20% rheumatic disease, 7% RP. Trigger factor: 73% cold, 8% stress, 8% exercise. Raynaud pattern: 31% single-phase, 53% two-phase, 16% three-phase. Clinical manifestations: livedo reticularis 66%, arthritis 34% and digital ulcers 16% in SRP; arthralgia 40%, perniosis 28%. Associated conditions: 7 MCTD, 8 JDM, 10 SLE, 3 SSc, 2 localized scleroderma, 1 CREST syndrome, 2 antiphospholipid syndrome (APS), 3 juvenile idiopathic arthritis, 1 Behçet’s disease. Auto-antibodies: ANA+ 48%, ENA+ 10%, DNA+ 7%. Capillaroscopy was performed in 92 patients, with mean follow-up time of 5,45 years. Out of 92 patients, 56 (61%) had NP, 14 (15%) NPP, 22 (24%) SPP. NP: 38 PRP, 9 SRP, 10 URP. NPP: 5 SRP, 9 URP. SPP: 20 SRP, 2 URP. Autoimmune rheumatic diseases with capillaroscopic pattern: SSc (1NP, 2 SPP), JDM (2 NPP, 6 SPP), SLE (4 NP, 2 NPP, 4 SPP), MCTD (1 NPP, 6 SPP), CREST (1SPP), APS (1 NP, 1 SPP). During the follow-up, 7 patients with NP changed to SPP (3 MCTD, 2 SLE, 1 CREST and 1 SSc). 54% were treated with transdermal nitroglycerine, 30% with nifedipine and 5% with bosentan.

## Conclusion

In our series we found a marked female predominance of RP, with a mean age of onset 10 years old. Compared with RP in the adulthood, we found a more frequent single- or two-phase pattern, and a higher association with systemic connective tissue diseases. These results are similar to those reported in other series.

## Disclosure of interest

None declared.

